# Supplementary material for: Molecular mechanism by which SARS-CoV-2 Orf9b suppresses the Tom70-Hsp90 interaction to evade innate immunity
Source: bioRxiv. 2025 Nov 18:2025.11.18.689095. Preprint. [Version 1] doi: 10.1101/2025.11.18.689095 (PMC12667937; doi:10.1101/2025.11.18.689095)
Supplement: Supplement 1 [file media-1.pdf]

Supplementary Information

Molecular mechanism by which SARS-CoV-2 Orf9b suppresses the Tom70-Hsp90 interaction to evade innate immunity

Noah Sherer[1], Abhishek Bastiray[1], Xiao-Ru Chen[1], Trivikram Molugu[1], Gaya P. Yadav[1], Tatyana I. Igumenova[1], Jae-Hyun Cho[1,*]

[1]Department of Biochemistry and Biophysics, Texas A&M University, College Station, TX 77843, United States

* Corresponding author: jaehyun.cho@agnet.tamu.edu

Figure S1

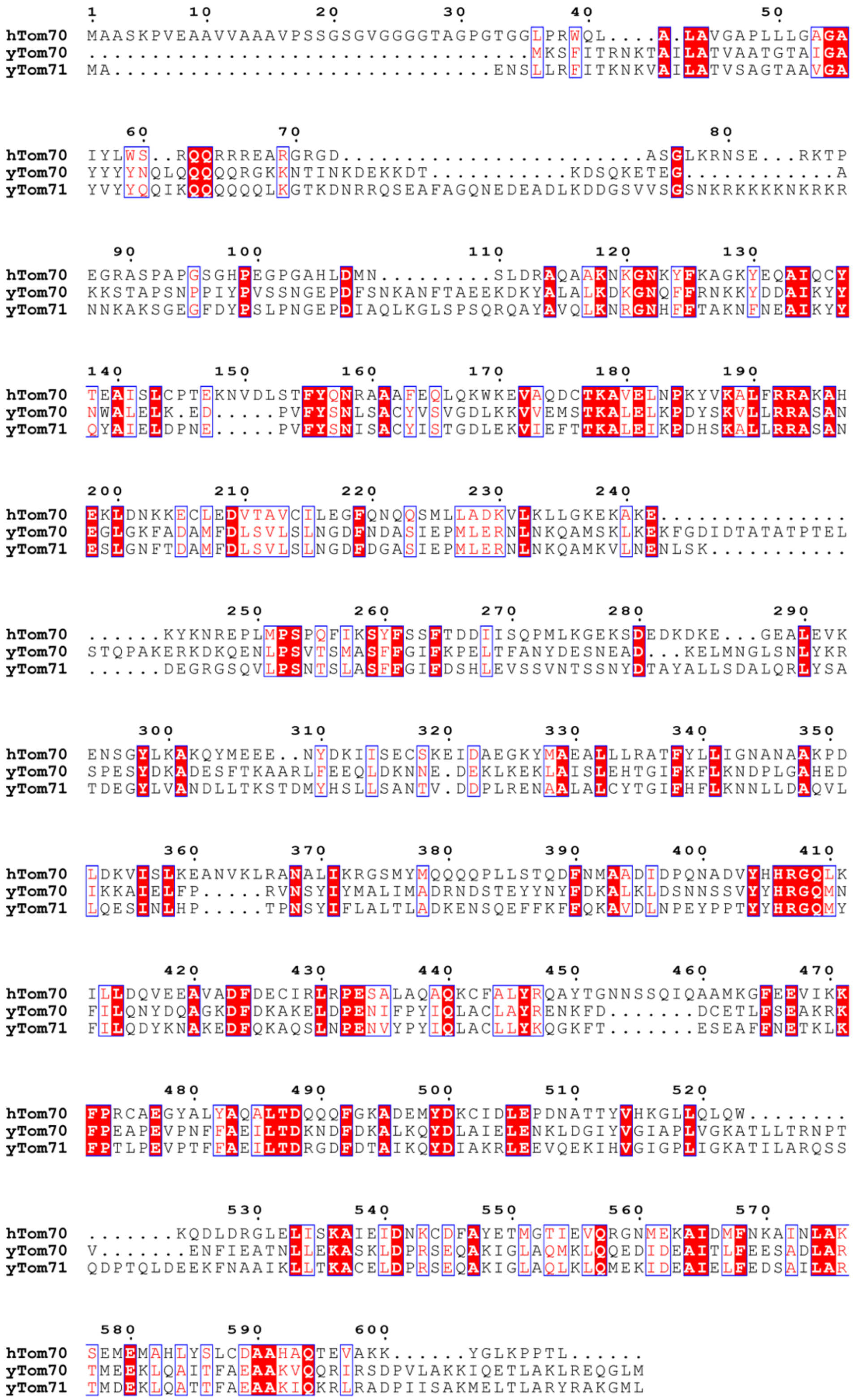

Supplementary Figure 1: Alignment of Human Tom70 to yeast Tom70/71. Multiple sequence alignment of Human Tom70 (UniProt ID: O94826), yeast Tom70 (UniProt ID: P07213) , and yeast Tom71 (UniProt ID: P38825) using the Clustal Omega webserver (https://www.ebi.ac.uk/jdispatcher/msa/clustalo) and displayed in Espript 3.0 (https://espript.ibcp.fr/ESPript/ESPript/index.php). Invariant residues are displayed with red background and conservative substitutions are displayed in red font. yTom70 has a 22% sequence identity (69% similarity) and yTom71 has a 21% sequence identify (68% similarity) with hTom70. There is an average of 20% identity (68% similarity) between all three sequences.

Figure S2

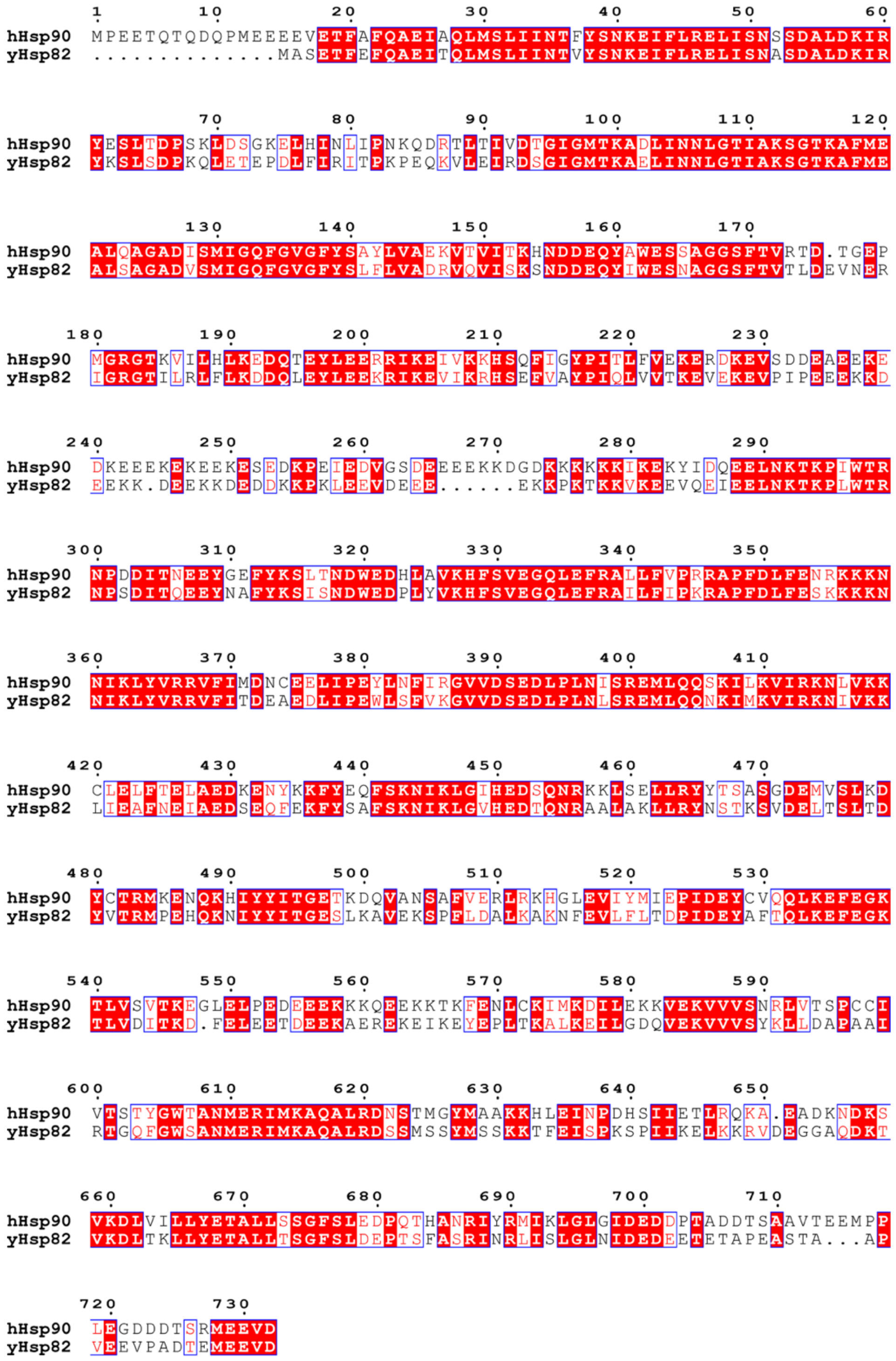

Supplementary Figure 2: Alignment of Human Hsp90 to yeast Hsp82. Multiple sequence alignment of Human Hsp90 (UniProt ID: P07900) and yeast Hsp82 (UniProt ID: P02829) using the Clustal Omega webserver (https://www.ebi.ac.uk/jdispatcher/msa/clustalo) and displayed in Espript 3.0 (https://espript.ibcp.fr/ESPript/ESPript/index.php). Invariant residues are displayed with red background and conservative substitutions are displayed in red font. On average, hHsp90 and yHsp90 share 60% sequence identity and 87% sequence similarity.

Figure S3

A

Hsp90 to Tom70

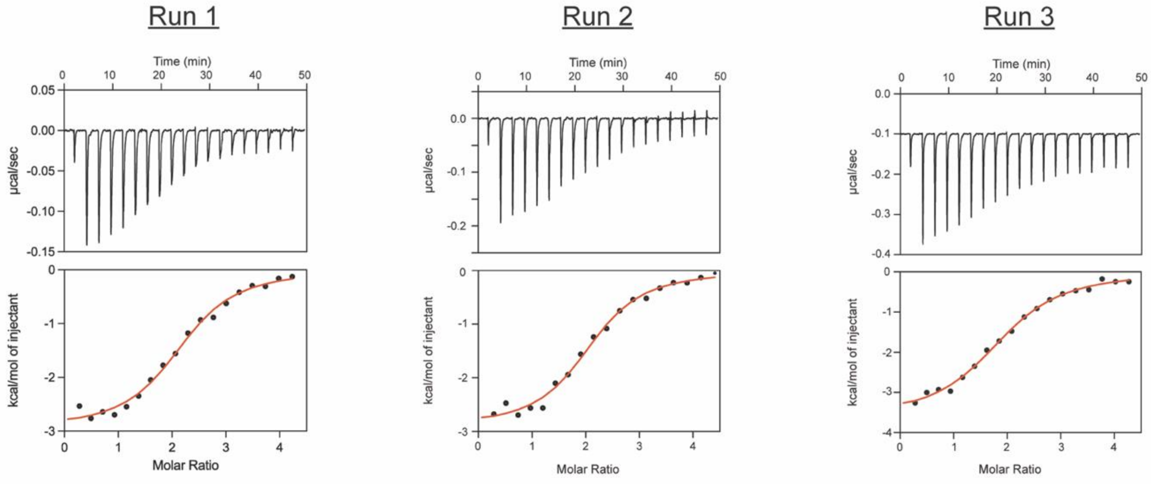

	Run 1	Run 2	Run 3	Average
**Stoichiometry (n)**	2.18 ± 0.07	2.08 ± 0.06	1.93 ± 0.05	2.06 ± 0.13
**K_D (µM)**	2.53 ± 0.65	2.41 ± 0.63	3.96 ± 0.76	2.97 ± 0.86
**ΔH (kcal/mol)**	-2.95 ± 0.18	-2.91 ± 0.16	-3.62 ± 0.22	-3.16 ± 0.40
**-TΔS (kcal/mol)**	-4.56 ± 0.22	-4.62 ± 0.22	-3.62 ± 0.05	-4.27 ± 0.56
**ΔG (kcal/mol)**	-7.51 ± 0.26	-7.56 ± 0.26	-7.25 ± 0.19	-7.44 ± 0.17

B

Hsp90 to Tom70:Orf9b

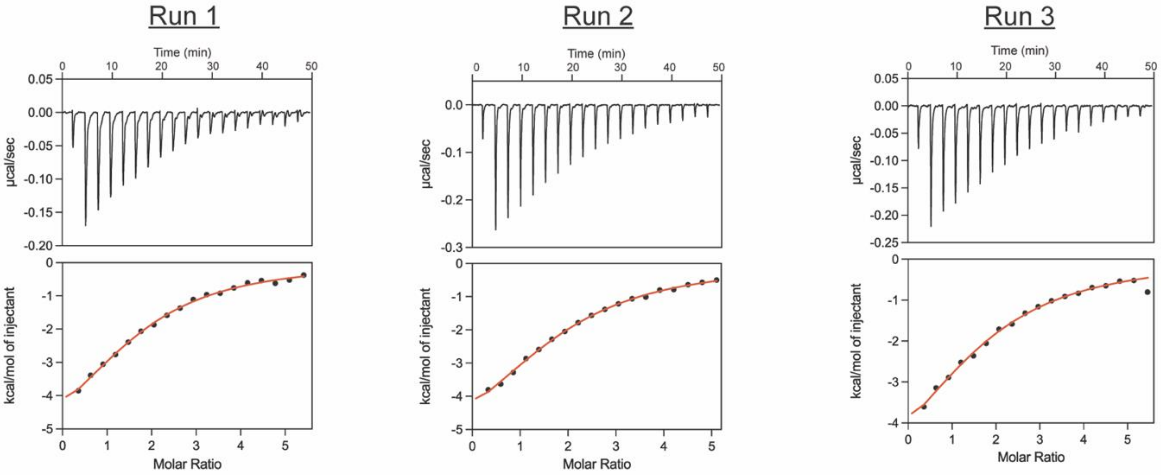

	Run 1	Run 2	Run 3	Average
**Stoichiometry (n)**	1.71 ± 0.08	1.79 ± 0.06	1.75 ± 0.09	1.75 ± 0.04
**K_D (µM)**	20.00 ± 4.56	25.60 ± 4.58	24.00 ± 5.87	23.20 ± 2.88
**ΔH (kcal/mol)**	-6.60 ± 0.99	-7.27 ± 0.81	-6.61 ± 1.02	-6.83 ± 0.38
**-TΔS (kcal/mol)**	0.29 ± 1.00	1.11 ± 0.82	0.42 ± 1.03	0.61 ± 0.44
**ΔG (kcal/mol)**	-6.31 ± 0.23	-6.16 ± 0.18	-6.19 ± 0.25	-6.22 ± 0.08

Figure S3 (continued)

C

Hsp90 to Tom70:Orf9b$_{Helix}$

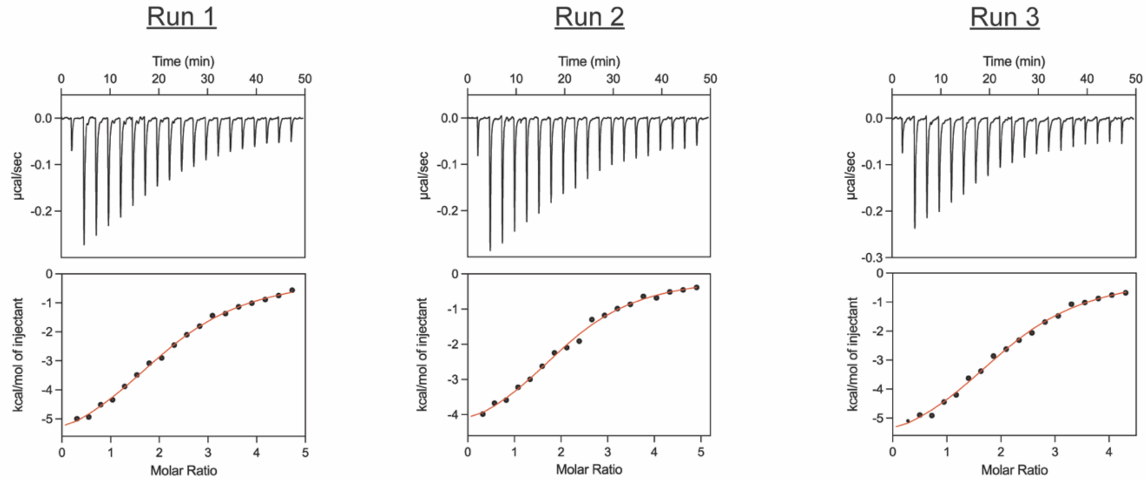

	Run 1	Run 2	Run 3	Average
**Stoichiometry (n)**	2.24 ± 0.07	1.8 ± 0.06	2.05 ± 0.08	2.03 ± 0.22
**K_D (µM)**	10.30 ± 1.78	7.24 ± 1.67	8.00 ± 1.75	8.51 ± 1.59
**ΔH (kcal/mol)**	-6.51 ± 0.42	-5.88 ± 0.48	-6.70 ± 0.52	-6.36 ± 0.43
**-TΔS (kcal/mol)**	-0.19 ± 0.44	-1.02 ± 0.49	-0.14 ± 0.54	-0.45 ± 0.56
**ΔG (kcal/mol)**	-6.69 ± 0.17	-6.90 ± 0.23	-6.84 ± 0.22	-6.36 ± 0.43

D

Hsp90 to Tom70:Orf9b ΔCDT (1-80)

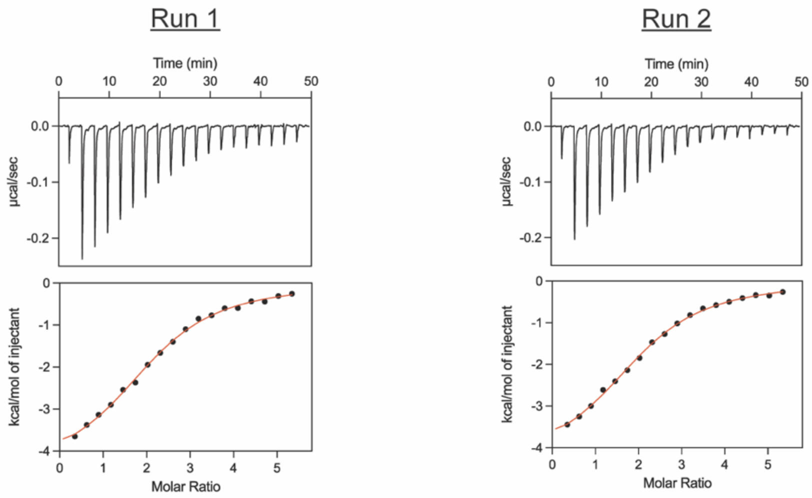

	Run 1	Run 2	Average
**Stoichiometry (n)**	2.13 ± 0.04	2.05 ± 0.04	2.09 ± 0.06
**K_D (µM)**	8.77 ± 1.23	9.27 ± 1.12	9.02 ± 0.35
**ΔH (kcal/mol)**	-4.52 ± 0.22	-4.4 ± 0.19	-4.46 ± 0.08
**-TΔS (kcal/mol)**	-2.27 ± 0.23	-2.35 ± 0.20	-2.31 ± 0.06
**ΔG (kcal/mol)**	-6.78 ± 0.14	-6.75 ± 0.12	-6.77 ± 0.02

Figure S3 (continued)

E

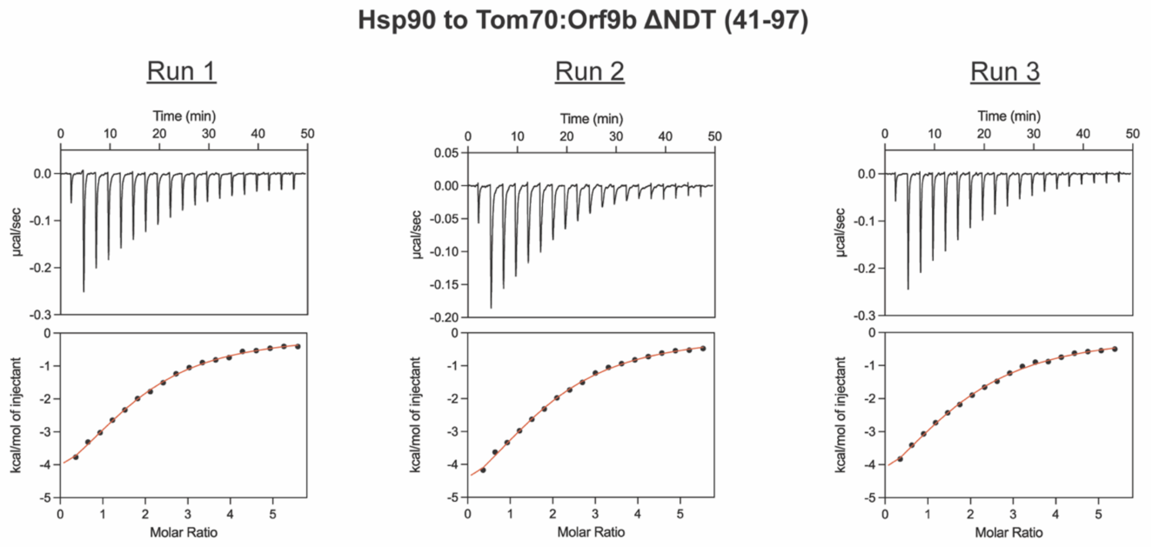

	Run 1	Run 2	Run 3	Average
**Stoichiometry (n)**	1.76 ± 0.05	1.81 ± 0.06	1.75 ± 0.06	1.77 ± 0.03
**K_D (μM)**	18.10 ± 2.71	19.10 ± 3.17	22.50 ± 4.15	19.90 ± 2.31
**ΔH (kcal/mol)**	-6.16 ± 0.51	-6.81 ± 0.62	-6.82 ± 0.77	-6.60 ± 0.38
**-TΔS (kcal/mol)**	-0.29 ± 0.52	0.48 ± 0.63	0.58 ± 0.78	0.26 ± 0.48
**ΔG (kcal/mol)**	-6.36 ± 0.15	-6.33 ± 0.17	-6.23 ± 0.18	-6.31 ± 0.07

Supplementary Figure 3: ITC thermograms, isotherms, and thermodynamic parameters for the interaction of full-length Hsp90 with **(A)** free Tom70, **(B)** Tom70:Orf9b complex, **(C)** Tom70:Orf9b$_{Helix}$ complex, **(D)** Tom70:Orf9b ΔCDT (1-80) complex, and **(E)** Tom70:Orf9b ΔNDT (41-97) complex. Numbers after ± symbol under each individual run represent fitting error. Numbers after ± symbol in the average values represent the standard deviation of the repeated runs.

Figure S4

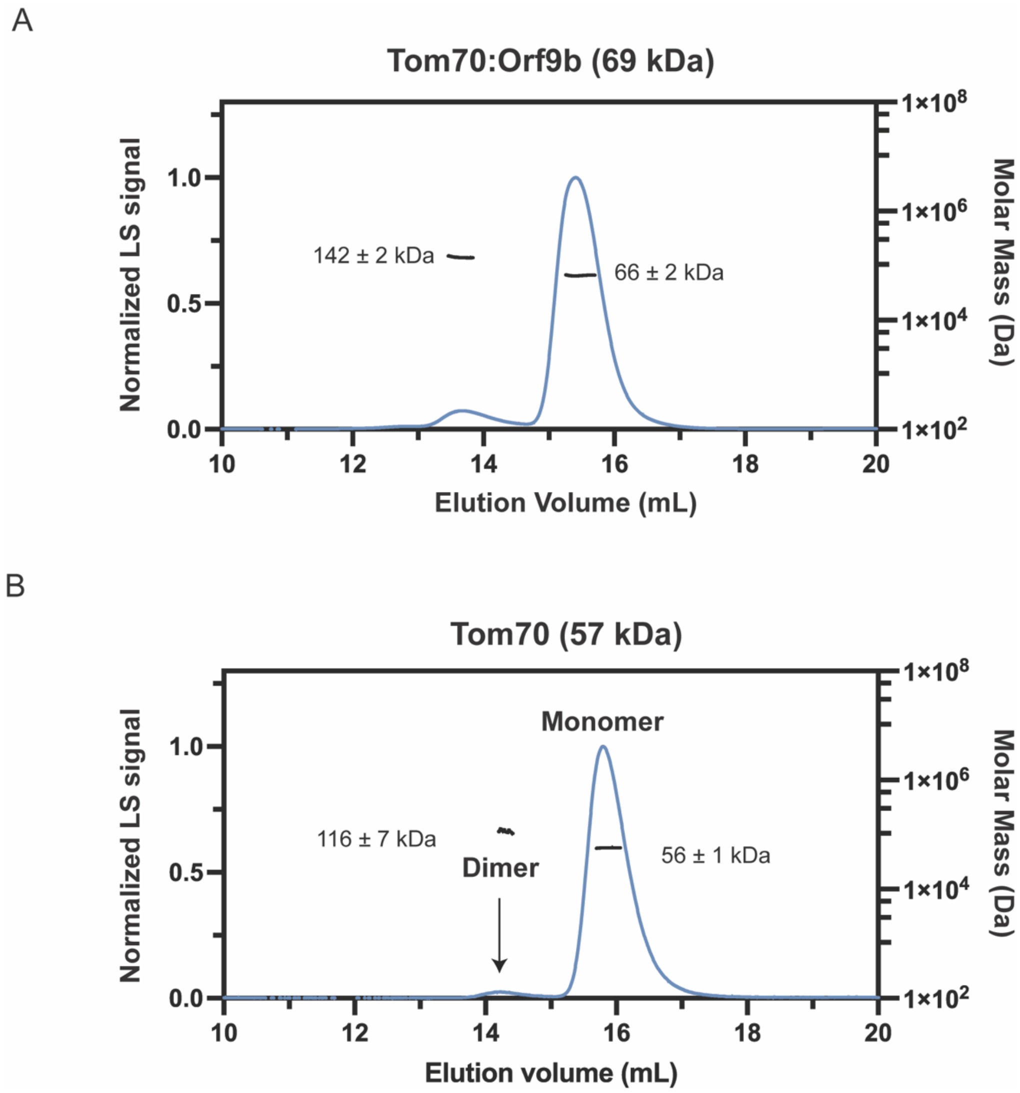

Supplementary Figure 4: SEC-MALS chromatogram of **(A)** Tom70-Orf9b complex and **(B)** free Tom70. The theoretical molecular weight of a monomer of Tom70 is 57 kDa and a dimer of Tom70 is 114 kDa. The theoretical molecular weight of Orf9b is 12 kDa making a 1:1 complex 69 kDa.

Figure S5

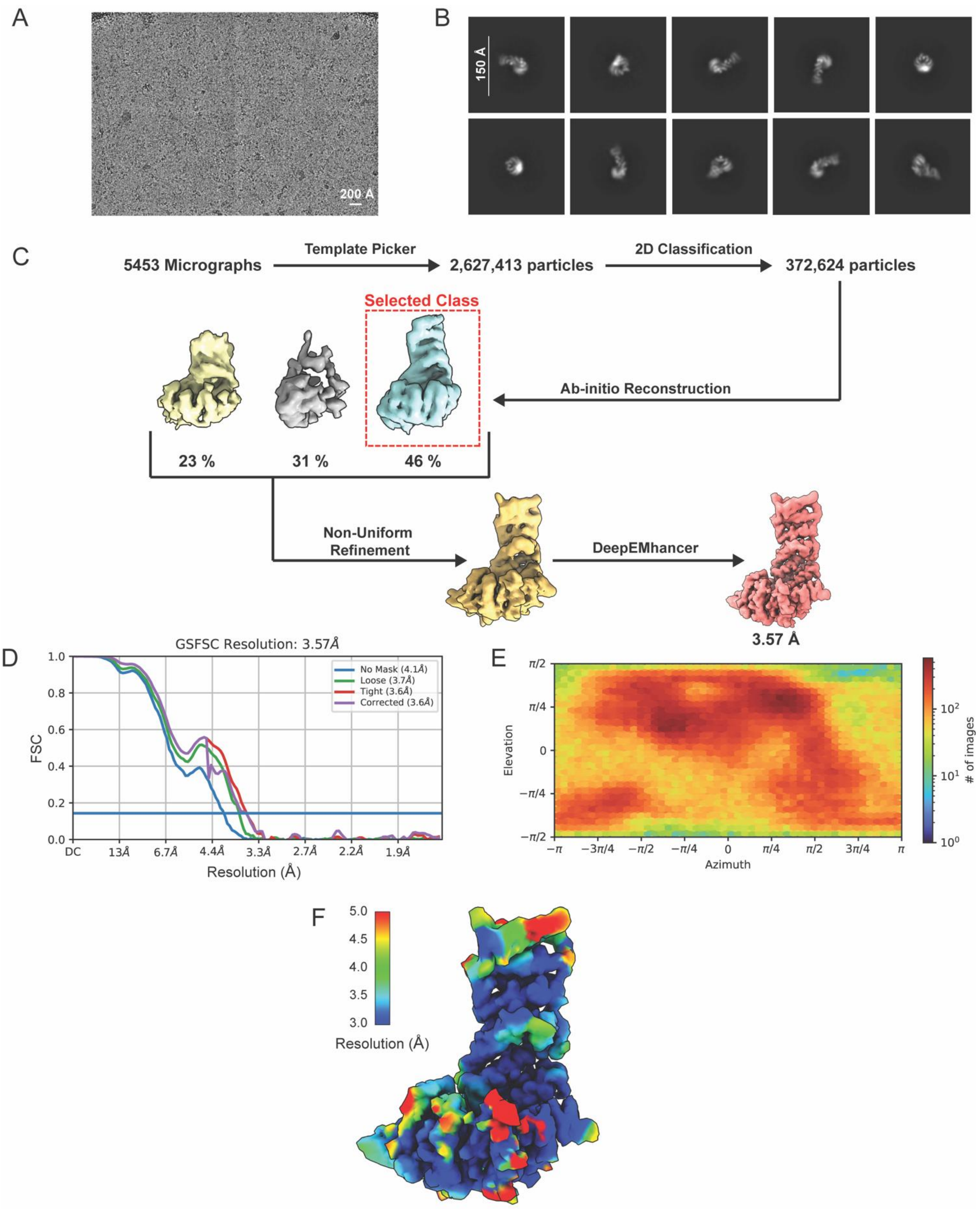

Supplementary Figure 5: (A) Representative micrograph, **(B)** representative 2D class averages, **(C)** data processing flow chart, **(D)** Fourier shell correlation (FSC) curves, **(E)** angular distribution, and **(F)** local resolution colored map of the cryo-EM derived Tom70 map.

Figure S6

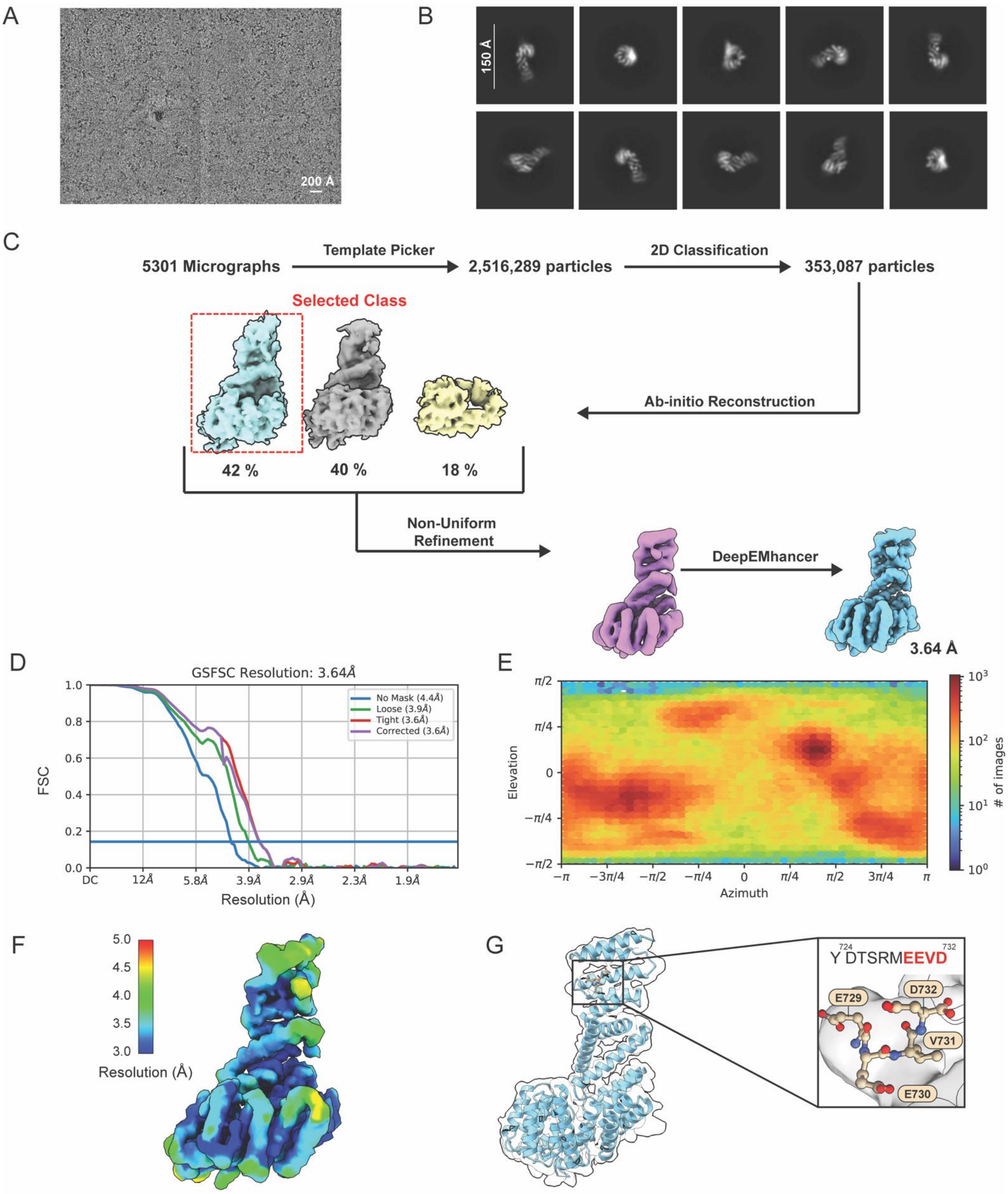

Supplementary Figure 6: (A) Representative micrograph, **(B)** representative 2D class averages, **(C)** data processing flow chart, **(D)** Fourier shell correlation (FSC) curves, **(E)** angular distribution, and **(F)** local resolution colored map of the cryo-EM derived Tom70:Hsp90$_{EEVD}$ complex map. **(G)** Only 4 of the 10 residues of the Hsp90$_{EEVD}$ peptide are visualized in the cryo-EM density.

Figure S7

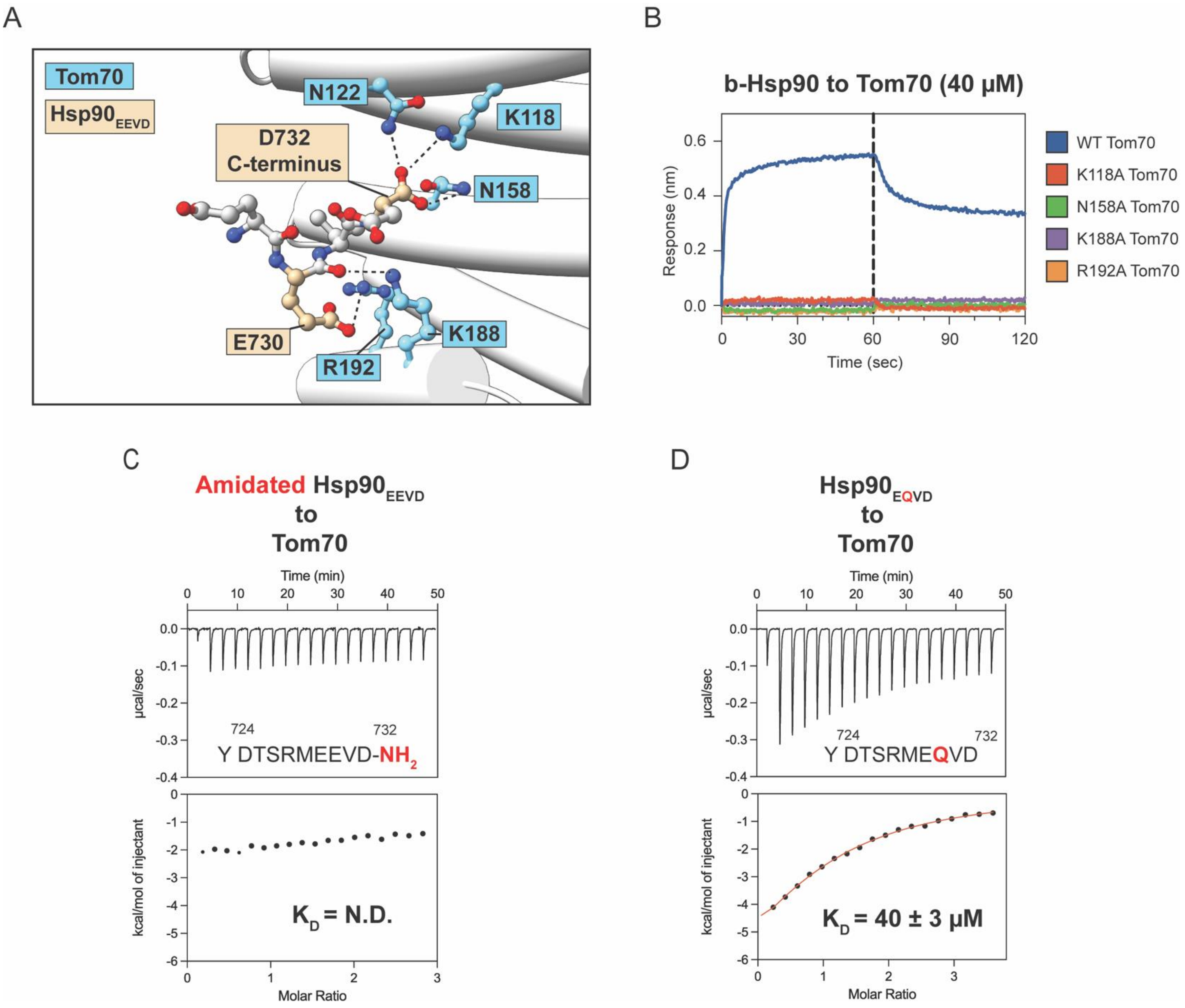

Supplementary Figure 7: (A) Interacting residues between Tom70 and $Hsp90_{EEVD}$ peptide. **(B)** BLI sensorgrams of biotinylated full-length Hsp90 assayed to Tom70 mutants. WT refers to “wild-type” Tom70. All Tom70 concentrations are at 40 µM. Dotted line demarcates the beginning of the dissociation phase. **(C-D)** ITC thermograms and isotherms for the interaction of Tom70 to an **(C)** amidated $Hsp90_{EEVD}$ peptide and a **(D)** $Hsp90_{EQVD}$ peptide. N.D. stands for “not-detected” due to the lack of sufficient heat generated.

Figure S8

A

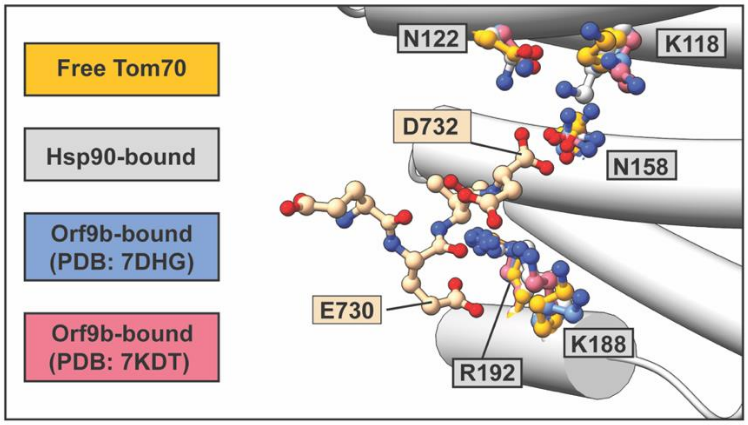

B

	Hsp90 bound		Free Tom70		Orf9b-bound (PDB: 7DHG)		Orf9b-bound (PDB: 7KDT)	
Residue	Backbone SASA (Å²)	Side Chain SASA (Å²)	Backbone SASA (Å²)	Side Chain SASA (Å²)	Backbone SASA (Å²)	Side Chain SASA (Å²)	Backbone SASA (Å²)	Side Chain SASA (Å²)
K118	0.89	45.67	0.22	52.39	0	33.80	0	55.85
N122	0	26.05	3.39	41.87	0.24	42.03	0.51	42.4
N158	4.35	27.92	3.68	24.73	1.29	26.58	0.79	34.67
K188	4.24	105.16	7.05	119.46	4.39	122.33	7.05	126.02
R192	3.10	43.06	2.25	47.17	0.18	47.42	0.19	43.51

C

Interaction Sites		Distance (Å)			
Tom70 residue	Hsp90 residue	Hsp90 bound	Free Tom70	Orf9b-bound (PDB: 7DHG)	Orf9b-bound (PDB: 7KDT)
K 118 NZ	D 732 OXT	3.26	7.33	6.14	6.72
N 122 ND2	D 732 OX	2.87	3.44	3.45	3.45
N 158 ND2	D 732 O	3.88	4.22	3.60	3.18
K 188 NZ	E 730 O	3.22	5.19	6.56	3.67
K 188 NZ	E 730 OE2	3.92	5.27	5.89	4.52
R 192 NH2	E 730 OE1	4.11	4.35	4.01	4.04

Supplementary Figure 8: (A) The side chains involved in $Hsp90_{EEVD}$ binding for free Tom70, $Hsp90_{EEVD}$ bound Tom70, and the two available structures of Orf9b-bound Tom70 are shown. The N-terminal region of each Tom70 was aligned (through matchmaker in ChimeraX) to the $Hsp90_{EEVD}$-bound form in order to calculate pairwise distance. **(B)** Calculated Solvent Assessable Surface Area (SASA) for each bound state of Tom70. SASA was calculated through the GETAREA software (https://curie.utmb.edu/getarea.html#:~:text=Sealy%20Center%20for%20Structural%20Biology,.edu%20or%20webraunATutmb.edu%20.). **(C)** Pairwise distance measurements for the interacting residues of Tom70 and Hsp90 in each bound state of Tom70.

Figure S9

A

$Hsp90_{EEVD}$ to Tom70

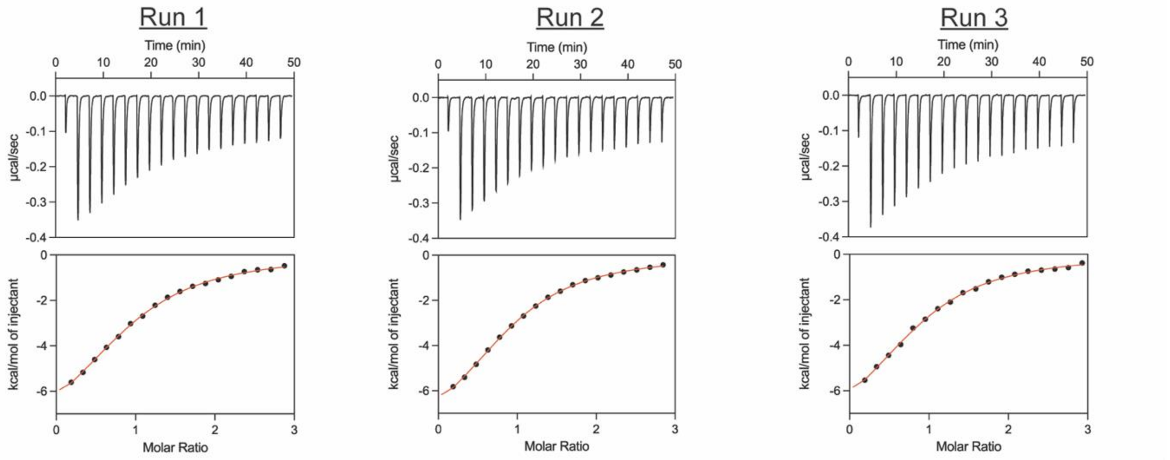

	Run 1	Run 2	Run 3	Average
**Stoichiometry (n)**	0.94 ± 0.02	0.92 ± 0.01	0.89 ± 0.02	0.91 ± 0.03
**K_D (µM)**	12.80 ± 1.43	10.90 ± 0.83	11.90 ± 1.65	11.87 ± 0.95
**ΔH (kcal/mol)**	-8.82 ± 0.50	-8.79 ± 0.32	-8.75 ± 0.63	-8.79 ± 0.04
**-TΔS (kcal/mol)**	2.26 ± 0.51	2.13 ± 0.32	2.14 ± 0.63	2.18 ± 0.07
**ΔG (kcal/mol)**	-6.57 ± 0.11	-6.66 ± 0.08	-6.61 ± 0.14	-6.61 ± 0.05

$Hsp90_{EEVD}$ to $Tom70{:}Orf9b_{Helix}$

B

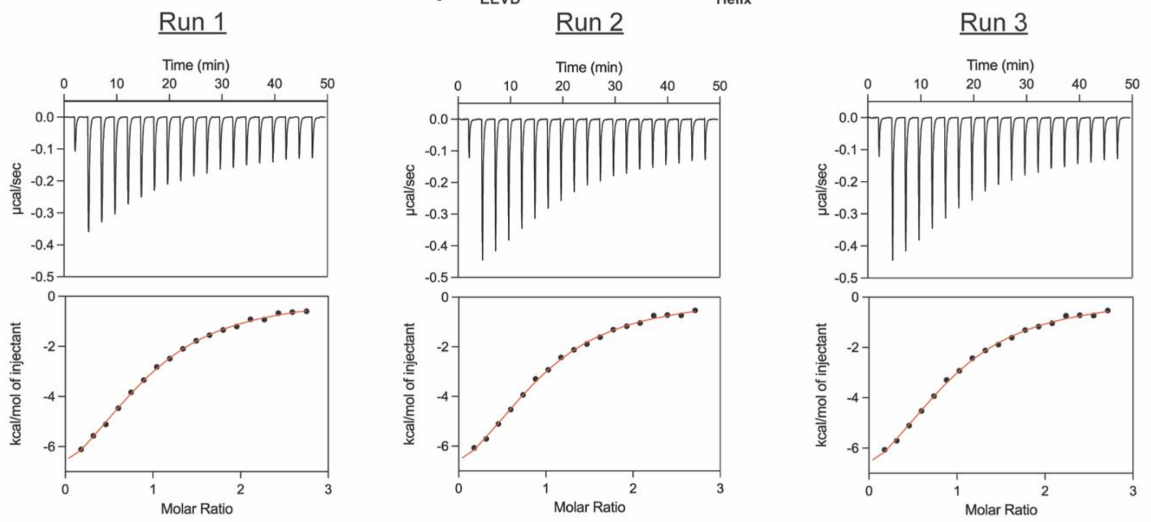

	Run 1	Run 2	Run 3	Average
**Stoichiometry (n)**	0.90 ± 0.02	0.90 ± 0.02	0.93 ± 0.03	0.91 ± 0.02
**K_D (µM)**	12.40 ± 1.46	11.80 ± 1.79	12.80 ± 1.68	12.33 ± 0.50
**ΔH (kcal/mol)**	-9.65 ± 0.58	-9.49 ± 0.71	-9.65 ± 0.73	-9.60 ± 0.09
**-TΔS (kcal/mol)**	3.07 ± 0.59	2.88 ± 0.72	3.09 ± 0.73	3.01 ± 0.12
**ΔG (kcal/mol)**	-6.58 ± 0.12	-6.61 ± 0.16	-6.57 ± 0.13	-6.59 ± 0.02

Figure S9 (continued)

C

Orf9b$_{Helix}$ to Tom70

Run 1 Run 2 Run 3

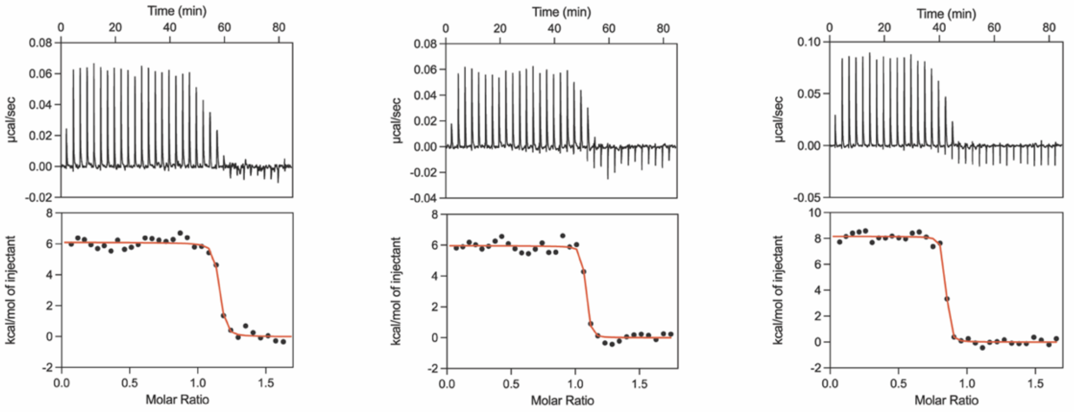

	Run 1	Run 2	Run 3	Average
**Stoichiometry (n)**	1.13 ± 0.01	1.06 ± 0.01	0.82 ± 0.01	1.00 ± 0.16
**K_D (nM)**	4.24 ± 1.88	2.03 ± 1.21	2.14 ± 1.06	2.80 ± 1.24
**ΔH (kcal/mol)**	6.10 ± 0.15	5.96 ± 0.13	8.16 ± 0.11	6.74 ± 1.23
**-TΔS (kcal/mol)**	-17.30 ± 0.30	-17.60 ± 0.37	-19.80 ± 0.31	-18.23 ± 1.37
**ΔG (kcal/mol)**	-11.20 ± 0.44	-11.70 ± 0.60	-11.60 ± 0.50	-11.50 ± 0.26

D

Orf9b$_{Helix}$ to Tom70:Hsp90$_{EEVD}$

Run 1 Run 2 Run 3

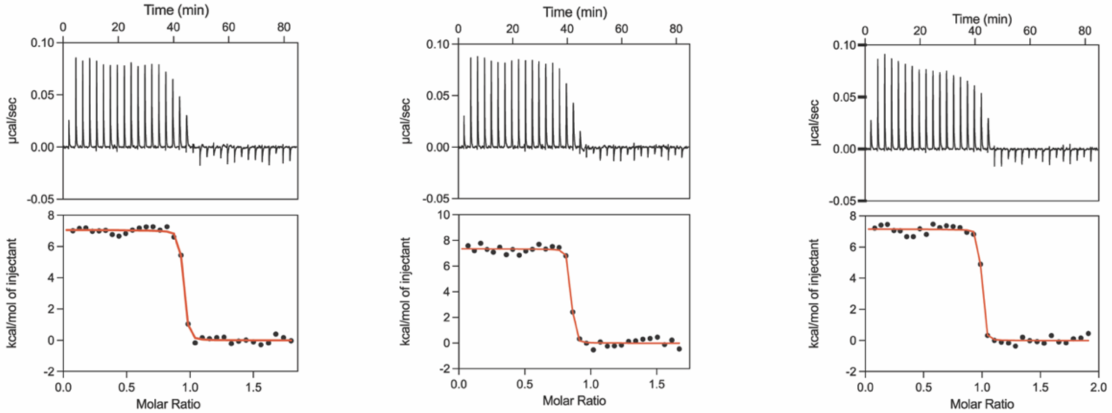

	Run 1	Run 2	Run 3	Average
**Stoichiometry (n)**	0.92 ± 0.01	0.83 ± 0.01	0.97 ± 0.01	0.91 ± 0.07
**K_D (nM)**	2.40 ± 0.07	1.44 ± 1.03	1.05 ± 0.08	1.63 ± 0.70
**ΔH (kcal/mol)**	7.05 ± 0.08	7.34 ± 0.11	7.15 ± 0.09	7.18 ± 0.14
**-TΔS (kcal/mol)**	-18.60 ± 0.08	-19.20 ± 0.43	-19.20 ± 0.10	-19.00 ± 0.35
**ΔG (kcal/mol)**	-11.60 ± 0.03	-11.90 ± 0.72	-12.00 ± 0.08	-11.83 ± 0.21

Supplementary Figure 9: ITC thermograms, isotherms, and thermodynamic parameters for the interaction of Hsp90$_{EEVD}$ with **(A)** free Tom70 and **(B)** Tom70:Orf9b$_{Helix}$ complex, as well as Orf9b$_{Helix}$ with **(C)** free Tom70 and **(D)** Tom70:Hsp90$_{EEVD}$ complex. Numbers after ± symbol under each individual run represent fitting error. Numbers after ± symbol in the average values represent the standard deviation of the repeated runs.

Figure S10

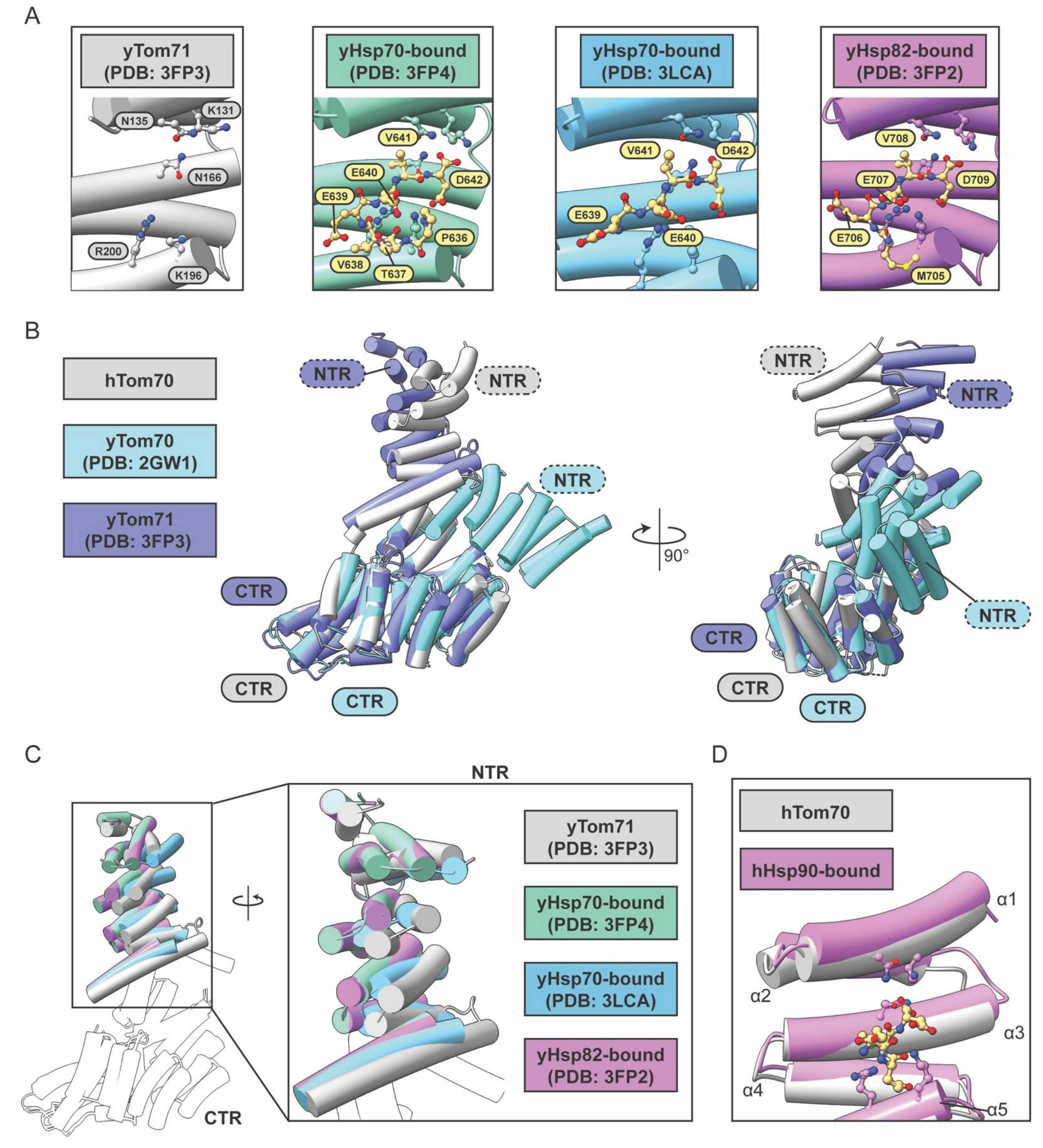

Supplementary Figure 10: (A) Comparison of the yHsp70/82 binding site in free yTom71 and all Hsp70/82-bound forms. **(B)** Structural alignment of free human (hTom70) and the two yeast Tom70 homologues (yTom70/71). The N-terminal Domain (NTD) and C-terminal Domain (CTD) are labeled for each. **(C)** All available structures of yTom71 bound yHsp70/82s aligned to free yTom71. The inset displays the NTD. **(D)** hHsp90-bound hTom70 aligned to free hTom70. Only helices α1-α5 are shown.

Figure S11

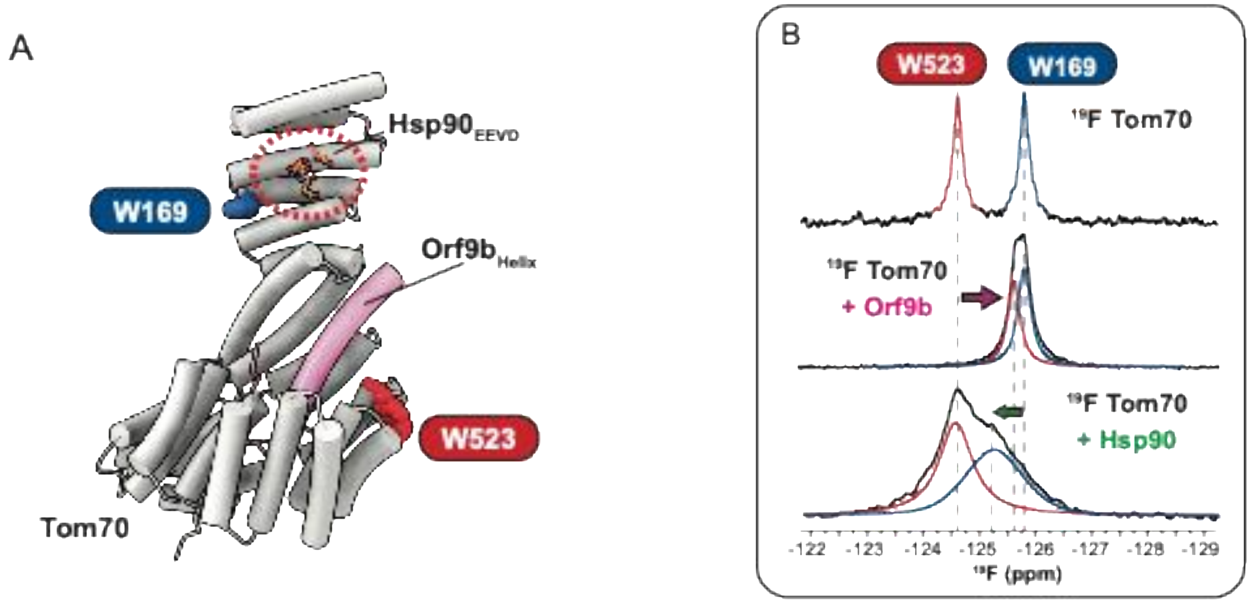

Supplementary Figure 11: (A) Schematic for ^{19}F Tom70. W169 (blue) is proximal to the $Hsp90_{EEVD}$ binding site, and W523 (red) is proximal to the $Orf9b_{Helix}$ binding site on ^{19}F Tom70. **(B)** ^{19}F Tom70 spectra for the binding full-length Orf9b (middle) and full-length Hsp90 (bottom) to Tom70.

Figure S12

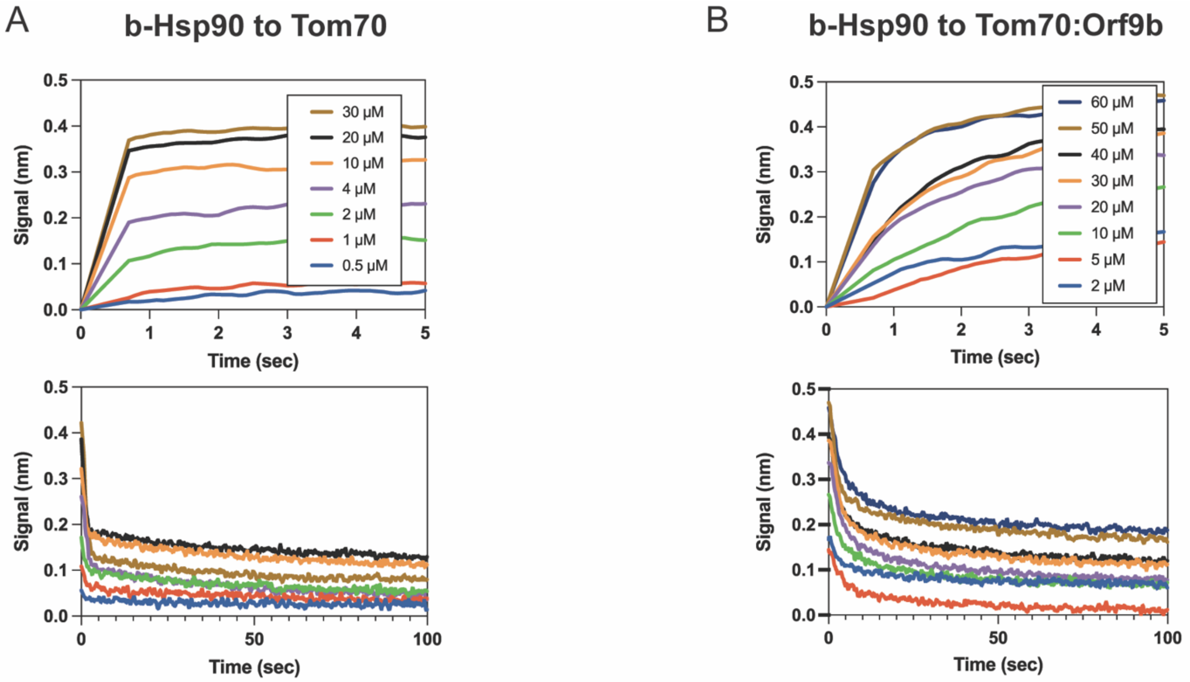

C

Ligand	Analytes	k_{off}^{BLI} (s^{-1})	K_D^{ITC} (μM)	k_{on} ($\mu M^{-1}s^{-1}$)
b-Hsp90	**Tom70**	1.39 ± 0.38	3 ± 1	0.46 ± 0.20
	**Tom70:Orf9b**	0.39 ± 0.07	22 ± 1	0.02 ± 0.01

Supplementary Figure 12: (A-B) Representative BLI sensorgrams of the association (top) and dissociation (bottom) of biotinylated Hsp90 (b-Hsp90) to **(A)** Tom70 and (**B)** Tom70 complexed with full-length Orf9b. (**C**) Table for BLI derived k_{off} values and calculated k_{on} values. k_{on} values were estimated by combining k_{off} values obtained from BLI with K_D values determined by ITC, i.e., $k_{on} = k_{off}^{BLI} / K_D^{ITC}$. Numbers after the ± represent propagated standard deviation.

Figure S13

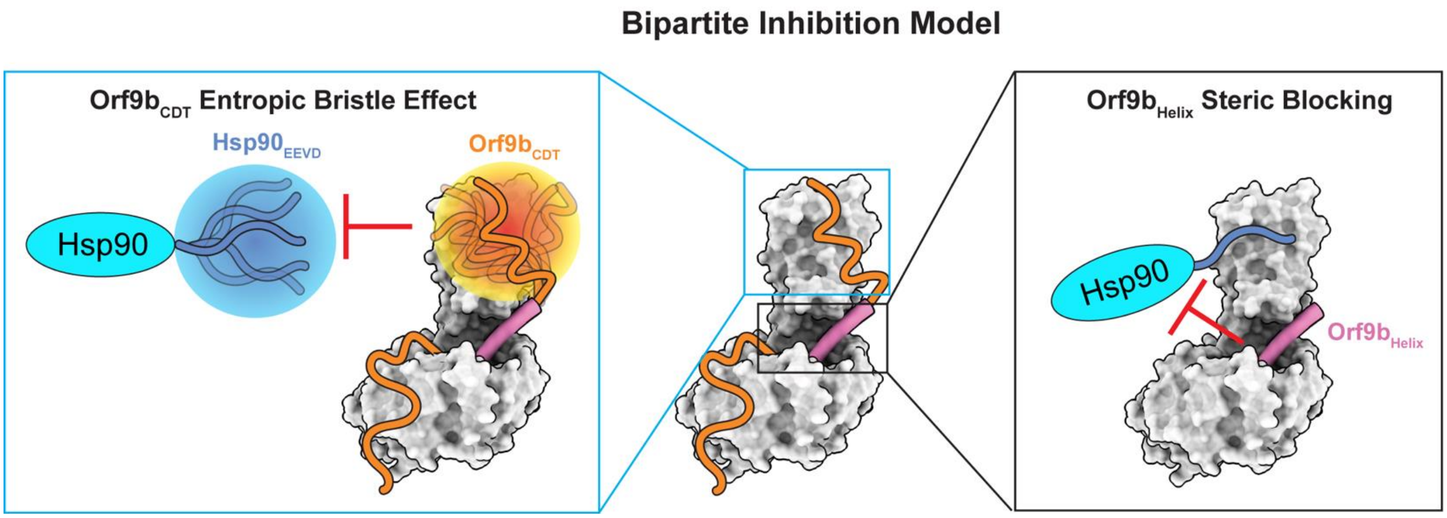

Supplementary Figure 13: Orf9b inhibits Hsp90 binding to Tom70 through a dual mechanism. The Orf9b C-terminal disordered tail (CDT) acts as an entropic bristle to sterically hinder the binding of $Hsp90_{EEVD}$ to Tom70. In addition, the $Orf9b_{Helix}$ sterically blocks the ancillary Hsp90 binding site. Orf9b-bound Tom70 (PDB 7DHG) is used to model the bipartite inhibition model.

Table S1

	Tom70 (EMD-73339) (PDB 9YQL)	$Hsp90_{EEVD}$-bound Tom70 (EMD-73359) (PDB 9YR4)
**Data collecting and processing**		
Magnification	105,000	
Voltage (kV)	300	
Electron exposure ($e^-/Å^2$)	50	
Defocus range (μM)	-0.8 to -2.4	
Pixel size (Å)	0.832	
Symmetry Parameters	C1	
Micrographs used	5453	5301
Initial Particle images (no.)	2627413	2516289
Final Particle images (no.)	372624	353087
Map resolution (Å) at FSC threshold 0.143	3.57	3.64
Map resolution range (Å)	3.0 to 6.5	2.5 to 5.5
Map post-processing	DeepEmhancer	
**Refinement**		
Initial model used	AlphaFold2	
Composition		
Non-hydrogen atoms	3794	3819
Protein Residues	476	479
B factors ($Å^2$)		
Protein	69.36/138.93/94.06	81.69/237.99/144.34
R.M.S.D from ideal values		
Length (Å)	0.004	0.004
Bond angles (°)	0.706	0.778
MolProbity score	1.02	1.03
Clashscore	2.38	2.5
Ramachandran plot (%)		
Favored	98.3	98.3
Allowed	1.7	1.7
Disallowed	0	0
CaBLAM outliers (%)	0.6	0.4
Rotamer outliers (%)	0.3	0.3
C-beta outliers (%)	0.0	0.0

Supplementary Table 1: Cryo-EM data collection, refinement, and validation statistics for Tom70 and $Hsp90_{EEVD}$-bound Tom70 maps and models.
